# Integrated Ecosystem Assessment: Lake Ontario Water Management

**DOI:** 10.1371/journal.pone.0003806

**Published:** 2008-11-25

**Authors:** Mark B. Bain, Nuanchan Singkran, Katherine E. Mills

**Affiliations:** Department of Natural Resources, Cornell University, Ithaca, New York, United States of America; University of Bristol, United Kingdom

## Abstract

**Background:**

Ecosystem management requires organizing, synthesizing, and projecting information at a large scale while simultaneously addressing public interests, dynamic ecological properties, and a continuum of physicochemical conditions. We compared the impacts of seven water level management plans for Lake Ontario on a set of environmental attributes of public relevance.

**Methodology and Findings:**

Our assessment method was developed with a set of established impact assessment tools (checklists, classifications, matrices, simulations, representative taxa, and performance relations) and the concept of archetypal geomorphic shoreline classes. We considered each environmental attribute and shoreline class in its typical and essential form and predicted how water level change would interact with defining properties. The analysis indicated that about half the shoreline of Lake Ontario is potentially sensitive to water level change with a small portion being highly sensitive. The current water management plan may be best for maintaining the environmental resources. In contrast, a natural water regime plan designed for greatest environmental benefits most often had adverse impacts, impacted most shoreline classes, and the largest portion of the lake coast. Plans that balanced multiple objectives and avoided hydrologic extremes were found to be similar relative to the environment, low on adverse impacts, and had many minor impacts across many shoreline classes.

**Significance:**

The Lake Ontario ecosystem assessment provided information that can inform decisions about water management and the environment. No approach and set of methods will perfectly and unarguably accomplish integrated ecosystem assessment. For managing water levels in Lake Ontario, we found that there are no uniformly good and bad options for environmental conservation. The scientific challenge was selecting a set of tools and practices to present broad, relevant, unbiased, and accessible information to guide decision-making on a set of management options.

## Introduction

Ecosystem-scale management is increasingly being initiated around the world to cope with complex problems spanning diverse environmental attributes over large areas. Methods to assess ecosystem management impacts and benefits are slowly developing through practice. Some notable US examples of ecosystem management are the landscape habitat modeling used for restoration of the Florida's Everglades [1], [2]; the indicator set used to track Chesapeake Bay management progress [3]; a key environmental tradeoffs comparison among scenarios for the Sacramento-San Joaquin Delta in California [4]; and long-term empirical monitoring of the Mississippi River [5]. In these and others cases, managed changes are expected to have numerous and widespread effects across many attributes of an ecosystem. Methods for anticipating and predicting magnitudes of change are needed to assess management options and identify a preferred alternative. Governments and decision-makers need concise and comparative information on their policy options, and the ecological science community should provide methods for forecasting ecosystem change [6], [7].

Assessing impacts of ecosystem scale change will commonly require a broad scope in space, ecosystem features of public interest, and different kinds of information. Nevertheless, the fundamental needs of impact assessment remain: quantitative estimates of effects on priority environmental resources under each proposed alternative [8]. We provide a method for comparing the environmental impacts of different policies for managing water levels in Lake Ontario. The governments of the United States and Canada determined [9] that the water management plan for this ecosystem needed re-evaluation and the potential effects on coastal environments were expected to be important and diverse [10]. We present a set of common assessment tools that can be used in concert to forecast diversified environmental impacts of different water level management plans. The tools are applied to the actual policy options under consideration to demonstrate our ecosystem impact assessment approach. The methods and results are sufficiently described to allow application to other ecosystem management programs.

## Methods

### Case Study and Area

Lake Ontario is the most downstream of North America's Great Lakes and it is positioned between Canada's Province of Ontario and the USA state of New York. Among the Great Lakes, it is the smallest (surface area of 18,960 km^2^) and second deepest (average depth of 86 m, maximum depth of 244 km) with the largest drainage area for its size (1∶3.4; watershed area of 64,030 km^2^). Nevertheless, a large majority (80%) of water input comes from Lake Erie through the Niagara River, and almost all water (93%) leaves by way of the St. Lawrence River [11]. The flow of water out of Lake Ontario is constrained by dams on the St. Lawrence River although lake level is affected by inflows, evapotranspiration, diversions, precipitation, and other hydrologic factors. Regulation of Lake Ontario water levels began in 1960 with a subsequent mean annual variation of 0.5 m (74.49–75.01 m, International Great Lakes Datum of 1985; IGLD 1985) [12]. However, seasonal variation in water level ranges from 0.3 to 1.1 m [13]. Previous to 1960, the lake had a greater range of water levels: 73.76–75.77 m.

The United States and Canadian governments adopted a treaty in 1909 establishing the International Joint Commission to manage binational waterways. In 1952 a set of water management rules was adopted by the International Joint Commission, and in 2000 the International Lake Ontario and St. Lawrence River Study Board was formed to re-evaluate options for St. Lawrence River discharge regulation. The Study Board [9] was assigned to consider environmental resources that were poorly assessed in the original water management plan and other important factors: economic costs, coastal erosion, commercial navigation, water supply, hydrology, hydroelectric power, tourism, and recreational boating. Any change in lake regulation would be the first substantial modification of Lake Ontario water management in a half century, and a new plan would likely remain in place for decades.

The coastal zone of Lake Ontario was the focus of water management impacts because the anticipated range of water level change was not expected to be important in the open waters of the lake [10]. The coastal zone includes a variety of water and shoreline formations that support a high diversity of species and a substantial portion of the flora and fauna of the Great Lakes [14], [15]. For example, a review [16] of the habitat requirements of 113 Great Lakes fishes reported that the vast majority of species used or required coastal habitats. Wetland vegetation of the bays and lagoons includes a high diversity of plants that are adapted to fluctuating water levels [17], [18]. Relative to the open lake, coastal bays and wetlands support high productivity that is enhanced by fluctuations in water levels [19], [20]. Finally, water level variations of Lake Ontario interact with the shoreline features to create complex patterns of coastal habitats [21].

The Study Board [9] identified a set of water management plans (Table 1) as policy alternatives for influencing water levels of Lake Ontario. Lake Ontario was first regulated in 1960 following dam building on the St. Lawrence River and the initial water regulation plan was labeled 1958A. Experience resulted in plan adjustments and the final operational plan used to the present is plan 1958D. Application of plan 1958D showed that adjustments, called deviations, were required at times to accommodate unusual or extreme conditions, and the actual record of actions was termed plan 1958DD (1958D with deviations). Numerous plans were developed and considered by the Study Board and five are used here: 1998, A+, B+, D+, and E. These address a range of interests (Table 1) consistent with the basic expectations of the International Joint Commission to enhance economic and environmental benefits. Finally, a plan was presented by the Study Board as a reference case: Plan E that depicts the unregulated conditions under the present ecosystem configuration and climate.

**Table 1:** Seven Lake Ontario water management plans defined by the Lake Ontario-St. Lawrence River Study Board [9].

Plan	Description and Purpose
**1958D**	The original plan used to set weekly outflows of Lake Ontario since 1963. The plan was developed using the 1860–1954 hydrologic record for Lake Ontario.
**1958DD**	Named for 1958D-with-deviations, this plan uses the decision criteria of actual water management used to set outflows of Lake Ontario since 1960s. Deviations were caused by situations such as winter ice formation and extreme hydrologic conditions outside the design criteria of Plan 1958-D.
**1998**	A new plan was proposed in 1998 to replace the Plan 1958D but was rejected by the International Joint Commission because it did not address issues on the environment and recreational boating. Plan 1998 was designed using contemporary hydrologic conditions to avoid many of the deviations that were being made from plan 1958D.
**A+**	This plan was designed to maximize overall economic benefits by striving for stable Lake Ontario water levels in a narrow range that matched desired conditions for a range of businesses.
**B+**	This plan was designed to simulate a more natural hydrological regime similar to conditions before lake regulation while minimizing impacts to other interests. The plan uses short and long term forecasts of water supplies in conjunction with the pre-project stage-discharge relationship to determine lake releases.
**D+**	Plan D+ was designed to increase the both economic and environmental benefits relative to plan 1958DD without significant losses to any other interests.
**E**	This plan was developed as the natural flow option with the current St. Lawrence River channel, dams, and structures. Considered best for the environment, this plan was designed to return the ecosystem to its pre-project state with enhanced condition of the flora and fauna.

### Impact Assessment

Our ecosystem impact assessment method was built with a set of established [22], [23] and commonly used impact assessment tools: checklists, classifications, matrices, representative taxa, and performance relations. A checklist was used to identify environmental attributes of interest for the assessment. Classification was used to organize the ecosystem into a series of shoreline classes with different physical properties. A matrix was used to identify the impact-sensitive combinations of environmental attributes and shoreline classes. Representative taxa were used to capture effects of lake level change by environmental attribute. Performance relations linked lake level change to representative taxa by shoreline class. Aside from these standard impact assessment tools, we introduced a somewhat novel concept to cope with the complexity of assessing ecosystem change at the scale of Lake Ontario: shoreline geomorphic classes in archetypal form. By an archetype we mean a model class of shoreline that exhibits defining features, typical characteristics, and distinguishing properties. Archetypes were not perfect representatives of a class, but they were used as a way to simplify the continuous range of variation seen in the biotic and abiotic environment.

Attributes of the environment were selected to address major biotic groups having substantial public and natural resource agency interest within to the coastal zone of Lake Ontario (Table 2). Environmental attributes were assembled from a list of indicator species proposed by the International Lake Ontario-St. Lawrence River Study [24]. Our list is longer and more specific but similar in being focused on prominent animals and plants in the coastal zone of Lake Ontario. Each biotic group was characterized by a representative taxa or assemblage that uses specific habitats; water level management impacts were assessed at this level. The orientation of water management policy-making around a broad array of biotic groups was established over many years of public debate on environmental implications of water level management on Lake Ontario.

**Table 2:** Environmental attributes for assessing impacts of water level regulation on Lake Ontario using management and public interests.

Biotic group	Habitats	Assessment taxa	Rationale for consideration in the assessment
**Plants**	Protected waters	Submerged aquatic vegetation	These plants stabilize sediments, reduce turbidity, and provide habitat for the spawning and rearing of young fish.
**Plants**	Protected shores	Emergent vegetation	Water edge plants that create habitat for benthic invertebrates, small forage fishes, juveniles of larger fish species, and nesting birds.
**Plants**	Land with saturated soil	Wetland vegetation	Plants in hydric soils above the water line form water key habitats for many organisms and are sensitive indicators of water levels and fluctuation regime.
**Invertebrates**	Littoral zone	Benthic invertebrates	Bottom dwelling organisms that are a major component of the aquatic food web and biodiversity.
**Fish**	Aquatic vegetation	Bowfin, *Amia calva*	Fish that use vegetated habitats throughout their lives or seasonally for reproduction, feeding, and refuge.
**Fish**	Wetland and submerged plants	Northern pike, *Esox lucius*	Spawning success often related to submerged and shallow water vegetation for eggs and larvae.
**Fish**	Tributaries	Rainbow smelt, *Osmerus mordax*	Spawning period water level changes can reduce habitats associated with stream inflows to the lake.
**Fish**	Littoral zone	Rock bass, *Ambloplites rupestris*	Dependent on shallow shoreline waters along open shores.
**Birds**	Protected waters	Mallard, *Anas platyhynchos*	Species dependent on submerged aquatic vegetation in still clear waters.
**Birds**	Emergent plants	Black tern, *Chlidonias niger*	Nesting in emergent vegetation can be disrupted by flooding, wave action, and water level change.
**Birds**	Marshes	King rail, *Rallus elegans*; Marsh wren, *Cistothorus palustris*	Nesting in marsh vegetation can be disrupted by flooding, wave action, and water level change.
**Birds**	Open shorelines	Bank swallow, *Riparia riparia*; Piping plover, *Charadrius melodus*; Killdeer, *Charadrius vociferous*	Species using open shoreline features such as sand, cobble beaches, and bluffs.
**Mammals**	Protected waters	Beaver, *Castor canadensis*	Dependent on protected shoreline waters and shores.

**Figure 1:**
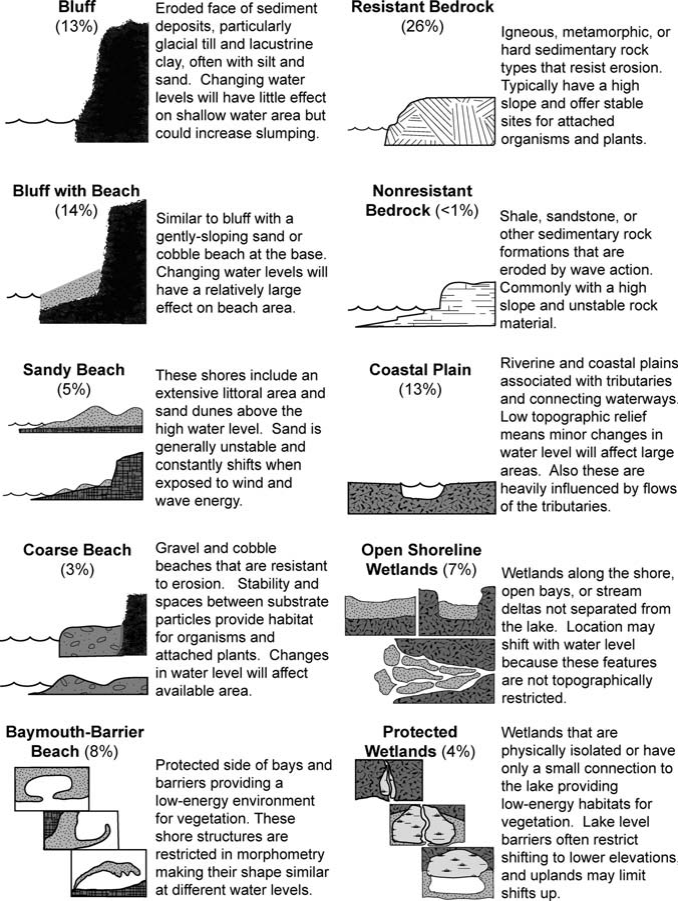
Shoreline classes for assessing lake level effects throughout the Great Lakes. The classes were identified, characterized, and illustrated by Stewart and Pope [25] and later refined and applied to all shoreline segments in the US and Canada. The percentage of the Lake Ontario shoreline composed of each class and its basic features relative to potential impacts are provided. Not included are artificial shorelines and other minor classes. Drawings included here were made from sketches in Stewart and Pope [25].

A coordinated US and Canadian effort to predict the consequences of varying water levels in the Great Lakes began in the late 1980s following a time when most of the lakes were at historically high levels. At that time a classification was developed [25] to provide a comprehensive description of Great Lakes shorelines that differed in sensitivity to water level changes. This classification system has been refined and applied throughout the Great Lakes [26] making it a standard for mapping and managing shorelines. The classification primarily relies on ten different shoreline types that differ in erosion rates, stability, and capability to adjust position with water level change. The characteristic features of these habitats are summarized in Figure 1 to define archetypes of each class. A quantitative assessment of ecological effects that may occur under different water level scenarios can be made at the full scale of any lake using data on the portion of the lake in each shoreline class.

A matrix of environmental attributes and shoreline classes was used to identify combinations for detailed assessment of water level change impacts. We considered each attribute and shoreline class in its archetypal form and how water level change would interact with distinguishing properties. This pair by pair review was used to reduce the potential range of impact analyses to those most likely to be important and worthy of further attention. Judgments on the likely significant impacts depended on the nature of water level change, how the shore class would respond to change, and how change could affect environmental attributes. Importance was judged by considering the likelihood that biotic changes symptomatic of ecosystem functional loss [27] would occur: reduction in sensitive species, community shifts toward tolerant organisms, unbalanced taxonomic composition, and food web alteration.

For each environmental attribute, one or more representative taxa were assessed by shoreline class using one or more relations between a water level change and impact magnitude. These models or performance relations were developed from scientific literature accounts of water level effects, and they were parameterized as changes in habitat quantity (e.g., percent change in area) or quality (suitability index; scale 0 for unsuitable and 1 as optimal). Performance relations spanned the range of water level change anticipated under the proposed water management plans.

**Figure 2:**
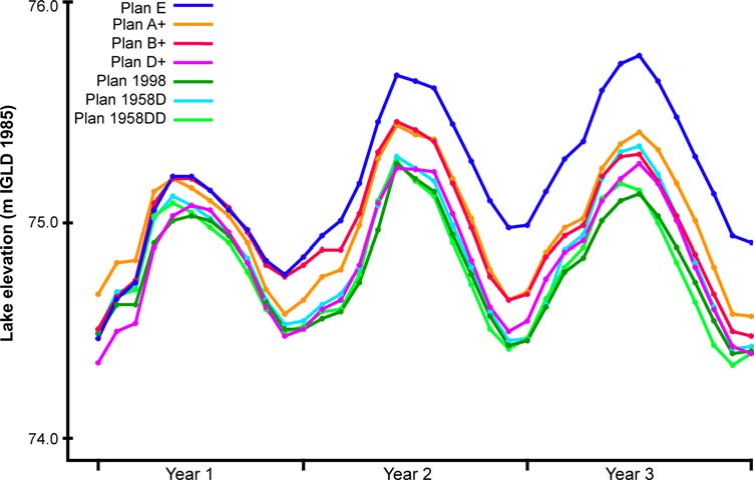
A sample 3-year sequence of monthly water levels under each water management plan. Simulated data were obtained from the Lake Ontario - St. Lawrence River Study Board [9]. Elevation in meters IGLD 1985 (International Great Lakes Datum of 1985) is the current standard that includes a baseline adjustment for glacial rebound in the earth's crust under the Great Lakes.

A 101-year water level sequence for each of the seven Lake Ontario water management plans (Table 1) was simulated using stochastic hydrologic inputs to the lake and the decision rules for each plan [9]. A 3-year portion of the simulated lake levels for each plan is shown in Figure 2. STELLA® version 8 [28] was used to model the effects of each of the seven 101-year simulated water management plans on the representative taxa. The relationship between each taxon and the change in water level caused by each plan was incorporated into the simulation model using step-linearly method and logical function (IF-THEN-ELSE). The 101-year simulation started with month 0 on January 1^st^ and ended at 1,212 months on December 31^st^. The interval of time between calculations was set to a small value of 0.0625 months to avoid artificial dynamics during the software calculation. The modeling values reflecting the taxa response to the change in water level under each of the water management plans were averaged by time period over the 101 simulated years. The results from the combination of representative taxa and water level change in each shoreline class were then rated by magnitude of expected impact from negative change (adverse impact), minor or equivocal impact, or a desirable change (favorable outcome). These ratings were assembled for all environmental attributes by water management plan to make comparisons of environmental consequences across all plans.

## Results

**Figure 3:**
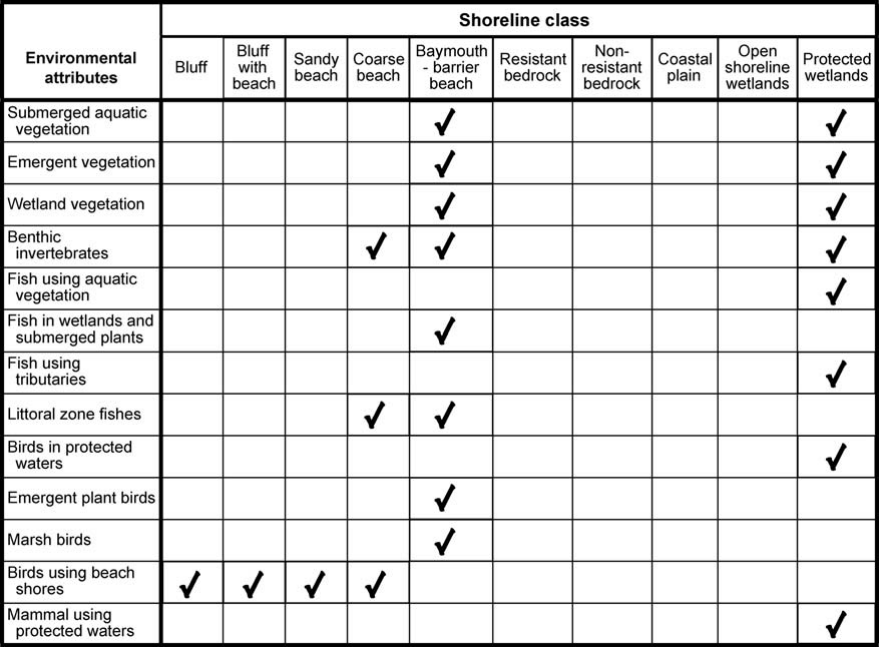
Matrix of environmental attributes and shoreline classes. Check marks indicate significant impacts were expected; empty cells indicate that no significant effect was anticipated for the range of water level changes being considered.

The review of potentially important impacts for 130 pairs of environmental attributes (13) and shoreline classes (10) resulted in a matrix (Figure 3) with most entries empty. Expected impacts were concentrated in two shoreline classes that have extensive shallow water and low sloping shores with limited physical response to water level change: baymouth-barrier beach and protected wetland classes. These two shoreline classes were similar in anticipated impacts with some environmental attributes covered in one shoreline class. Impacts on plants were included under both shoreline classes and were found below to be similar in separate analyses. This first analysis step also revealed that birds using shorelines with beaches and bluffs were expected to be affected by water level change across a set of shoreline classes. Finally, four classes of shorelines were not designated to have significant impacts from water level changes at the scale of variability predicted for the water management plans. These shoreline classes were either steep with immobile rock (resistant bedrock, nonresistant bedrock), habitats expected to physically relocate with water levels (open shoreline wetland habitats), or were already highly variable in levels due to tributary flows (riverine and coastal plain). Water level changes in Lake Ontario will have little effect on the areal extent of these habitats or taxa found in them. From the data on amounts of shoreline in each class (Figure 1), the matrix analysis identifies about half the shoreline of Lake Ontario as potentially sensitive to water level change with a minor portion (12%) being highly sensitive.

### Bluffs

Coastal bluffs experience erosion and recession from wave action on their face (Figure 1), and changes in water level would alter bluff erosion and recession rates in the short term. A decline in mean lake level would reduce erosion, while a rise in lake level would increase erosion until an equilibrium profile returns [29]. When waves erode the base of a bluff, its slope angle increases and the base becomes unstable, which may result in mass movements of material [30]. Horizontal recession rates of Lake Ontario bluffs range from 0.3–1.5 m/yr [31], and the rate changes with lake level [32]. Consequently, slumping and caving of overlying material would increase as water levels rise and decrease as levels fall.

**Figure 4:**
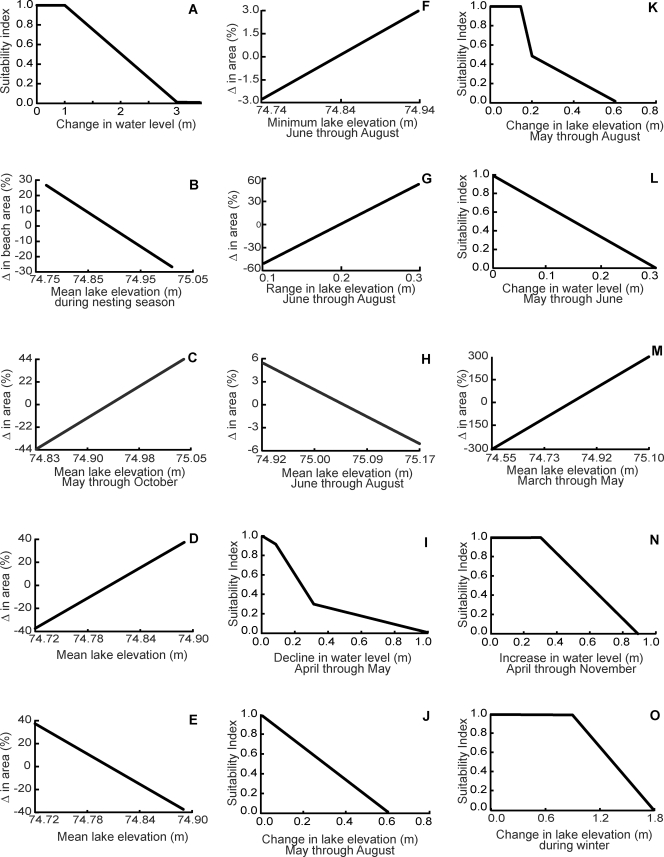
Relationships between Lake Ontario water level and environmental attributes. A) bank swallow nesting in bluff habitat, B) piping plover nest area on sand beaches, C) benthic invertebrate habitat along coarse beaches, D) rock bass habitat along coarse beaches, E) killdeer foraging and nesting area on coarse beaches. Relationships for both baymouth-barrier beach shorelines and protected wetland and backwater shoreline classes: F) area of submerged aquatic vegetation, G) area of emergent vegetation, and H) area of wetland vegetation. Baymouth-barrier beach shoreline relations included: I) suitability of habitat for Northern pike embryos and the earliest fry stages, J) nesting suitability for black tern, and K) nesting suitability for king rail. Relations only for the protected wetland and backwater shoreline classes were: L) suitability of habitat for bowfin early life stages, M) area of rainbow smelt adult staging and early life rearing habitat, N) nesting suitability for marsh wren, and O) suitability of habitat for overwintering beaver.

A rise in water levels and an increase in erosion of the bluff can be detrimental to shoreline birds that nest in bluffs, such as bank swallows (*Riparia riparia*). Bank swallows are found throughout the Great Lakes region in summer (June to August). They construct nests at the end of a tunnel (60–95 cm long) near the top of coastal bluffs [33]. The length of nest tunnels is equal to the common horizontal recession rate of bluffs, and any increase in bluff recession rate would jeopardize survival of eggs or young. An increase in water level greater than 1 m is expected to elevate bluff recessions and decrease nesting suitability of bank swallows: conditions will become unsuitable when the water level increases 3 m or more (Figure 4A). This relation is very similar to that measured for bank swallow nesting along the Sacramento River, California [34]. Comparing the results among the seven water management plans indicate plan 1958DD provided the most suitable conditions (suitability = 0.93) for nesting of bank swallows, whereas plan E had the lowest but still high score (0.82). All other plans were intermediate in suitability resulting in favorable bank swallow nesting conditions for all plans (Table 3).

**Table 3:** Impact ratings of water regulation plans on each representative taxa in each shoreline class.

Representative taxa	Shoreline class*	1958D	1958DD	1998	A+	B+	D+	E
**Bank swallows**	Bluff	**+**	**+**	**+**	**+**	**+**	**+**	**+**
**Piping plovers**	Bluff with beach, sandy beach	**−**	o	o	**−**	o	o	**−**
**Benthic invertebrates**	Coarse beach	**+**	**−**	**−**	o	**−**	**−**	**+**
**Rock bass**	Coarse beach	o	**−**	o	o	o	o	**+**
**Killdeer**	Coarse beach	o	**+**	o	o	o	o	**−**
**Submerged aquatic vegetation**	Baymouth-barrier beach, protected wetlands	**+**	o	o	**+**	o	o	**+**
**Emergent vegetation**	Baymouth-barrier beach, protected wetlands	o	**−**	**−**	o	**−**	**−**	**+**
**Wetlands**	Baymouth-barrier beach, protected wetlands	o	**+**	o	o	**+**	o	**−**
**Northern pike**	Baymouth-barrier beach	o	**+**	**+**	o	o	o	**−**
**Black tern**	Baymouth-barrier beach	**+**	**+**	**+**	**+**	**+**	**+**	**+**
**King rail**	Baymouth-barrier beach	**+**	**+**	**+**	**+**	**+**	**+**	**−**
**Bowfin**	Protected wetlands	**+**	**+**	**+**	**+**	**+**	**+**	**+**
**Rainbow smelt**	Protected wetlands	**+**	o	o	**+**	**+**	**+**	**+**
**Mallard**	Protected wetlands	o	**+**	**+**	o	**+**	**+**	o
**Marsh wren**	Protected wetlands	**+**	**+**	**+**	**+**	**+**	**+**	o
**Beaver**	Protected wetlands	**+**	**+**	**+**	**+**	**+**	**+**	**+**

Impact rating symbols: **−** for adverse impact, **o** for a minor or equivocal impact, **+** for a desirable change (favorable outcome).

* Note that multiple shoreline classes are shown in four table rows.

### Bluffs with Beach and Sandy Beach

Gently sloping sand beaches with or without bluffs (Figure 1) will be inundated or exposed with changes in water levels. We expect that beach area during shorebird nesting and summering can serve as a measure of the nesting habitat extent for some birds. Piping plover (*Charadrius melodus*) is an endangered species and beach nesting bird [35], [36] found along Lake Ontario from March through August. Nests are maintained for four to five weeks while eggs incubate and several additional days are necessary for the young to fledge [37]. The low slope and constrained width of sand beaches make nesting habitat vulnerable to changes in water level. For example, on a beach with a slope of 0.015 (the average beach slope for Lake Ontario [29]), over 67 m of beach width is lost if water level rises by 1 m. We assume that the width of an archetype beach is about 30 m. Thus, 8.0 m of beach gain or loss will result in the range of change in the beach area for piping plover nesting between −27 and 27% (Figure 4B). The simulated water levels and changes in beach area ranged from a monthly area loss of 81% to a gain of 28%. Again, water management plan 1958DD provided the best conditions (mean loss of 11% beach area) and plan E the worst (59% mean loss). Four plans provided minor beach area losses and were consider equivocal in impact while three plans were predicted to result in large area losses and adverse beach nesting conditions (Table 3).

### Coarse Beach

Coarse beaches are composed of rocky material, typically with particle sizes of gravel to cobble, that are resistant to wave action and do not move readily with water level changes (Figure 1). Shallow and turbulent waters with coarse material provide habitat for a variety of invertebrates and fish [38], [39], and the exposed coarse beaches support some birds. Three taxa were used for assessing water level change in coarse beach habitat: benthic invertebrates (e.g., insects, mollusks, and crustaceans), rock bass (*Ambloplites rupestris*, shallow rocky habitat fish), and killdeer (*Charadrius vociferous*, shore zone bird). The linear relationship between the change in area of coarse substrate littoral area and exposed beach were developed for each taxon.

Water level changes are expected to affect invertebrates by altering the total amount of submerged habitat available and shifting the location of wave action within this habitat. During the period of rapid growth and reproduction (May through October), high water levels will expand the productive area while lower water levels will contract the available shallow-water area with coarse substrate. Average water level in Lake Ontario from May through October was 74.94±0.1 m with a coarse beach slope of approximated 0.0075 (half the slope of sandy beach [29]). An increase of mean water level by 0.1 m will increase the area available to benthic invertebrates by 13 m, and with a 30-m wide coarse beach zone the usable area would change by 44% (Figure 4C). Under all water plans the area of invertebrate habitat increased in early summer (maximum 142%) and declined (maximum 188%) afterward with falling lake levels. Over the period, water management plans E and 1958D increased invertebrate habitat (42% and 3% respectively) and were considered favorable (Table 3). Plan 1958DD had the most negative (−53%) predicted invertebrate habitat change, and was considered adverse with three other plans of similar outcome. One plan (A+, Table 3) had an expected minor loss of habitat and was considered equivocal in impact. Using the same calculations on an annual basis, a similar magnitude of habitat change was found for fish (Figure 4D) and again plan E provided the largest increase (81%) and plan 1958DD the largest loss (−26%) in habitat. These plans were classified as favorable and adverse (respectively, Table 3) with the other plans displayed minor gains and losses that were considered equivocal in impact. Fish that inhabit the littoral zone of coarse beaches forage among the interstitial spaces of the substrate to find invertebrates. Our representative fish for this habitat was rock bass, a species that commonly inhabits rocky areas in shallow waters of northern lakes year round, and spawns in these habitats during the spring [40].

The killdeer is a shoreline bird that forages and nests on coarse beaches. This species migrates from wintering habitats in southern North America to breeding grounds in the Great Lakes region [41] as early as March, nest in shallow depressions, and feed almost entirely on invertebrates by dabbing the ground or gravel [42]. Their beach habitat area is largely determined by the mean water level. The change in beach area with change in water level was estimated in the same way as for rock bass, but usable habitat varied in an inverse manner (Figure 4E). Exposed beach area for killdeer increased the most with plan 1958DD (26%) and declined the most with plan E (−81%) causing these plans to be classified as favorable and adverse (Table 3). Again, the other plans displayed minor gains and losses that were considered equivocal in impact. Essentially, changing water levels directly tradeoff habitat available to rock bass and killdeer because the beach-littoral zone slope is a constant and both species use habitats either above or below the water surface.

### Baymouth-Barrier Beach

The baymouth-barrier beach shoreline class provides protected, low-energy sites supporting abundant submerged aquatic vegetation, emergent plants, and wetlands. Because of the diversity of vegetated zones, baymouth-barrier habitats are used by a large variety of biotic groups, either permanently or during discrete times of the year. As such, these areas support some of the richest plant and animal communities, including species that are particularly important ecologically and others that are valued by humans. Baymouth-barrier beach shorelines are restricted in morphometry and are unable to change shape or move with changing water levels (Figure 1). A lowering of water level will reduce open water habitat area, reduce the length of land-water margin, and increase intermittently flooded land area because these sites are confined on the protected side. An increase in water level will expand the wetted habitat area, increase the length of margins, and reduce low-lying upslope area.

Lake level fluctuations strongly influence the extent, location, and density of aquatic vegetation [18], [43]–[45]. The areal coverage of submerged and floating-leaved aquatic vegetation (SAV) is determined by the potential habitat space available between the minimum water level and the depth of light penetration (i.e., the photic zone). For baymouth-barrier beach shorelines, the area of SAV declines as water levels fall and increases as water levels rise because gains in habitat upslope exceed downslope loss. SAV cover was mapped in an archetype baymouth-barrier beach site (Blind Sodus Bay [46]) and dimensions on 10 transects across the photic zone showed an average 151-m width of vegetation cover and an average slope of 0.024. This approximates the photic range (up to 4.5 m) predicted for SAV growth [47] for a Lake Ontario waters with a secchi disk transparency of 3.5 m [48]. The maximum SAV depth was also near the maximum available water depth inside a baymouth-barrier beach site. During lake level regulation, the average minimum water level during the SAV growing season (June through August) was 74.84 m. A variation in lake level during this period of 0.1 m would be expected to alter the available area for SAV (Figure 4F). Plan E provided the largest positive change (7%) and was similar to plans 1958D and A+; these plans were considered favorable for SAV coverage (Table 3). Plan 1958DD had the smallest average change in SAV habitat (<2% increase) and was considered equivocal in impacts on SAV coverage. The same rating was applied (Table 3) to three other plans with minor predicted change in SAV cover. No plan was considered adverse to SAV growth because none had a sizable predicted loss in coverage.

Emergent vegetation inhabits the shallow water zone between open water and wetlands. The habitat of emergent vegetation is defined by the variability between mean and minimum water levels [18] during the growing season (June–August). We assume a change in the range of water level variability changes suitable habitat area in a direct linear manner. Since regulation, the average mean to minimum range has been 0.195 m along a slope of 0.024 from the characterization of Blind Sodus Bay. Therefore, the fluctuation range of 0.195 m alternately floods and dewaters an average of 8.12 m of substrate that supports an emergent vegetation fringe between wetlands and open water. With increases in the fluctuation range, the emergent vegetation fringe will expand while decreases in the water level range will reduce the area of this habitat. For a range of 0.3 m, the emergent zone will encompass 12.71 m, an increase of 56%. If the range declines to 0.1 m, emergent vegetation will be limited to a 4.21 m band, a decline of 48% from the present extent (Figure 4G). The two plans bracketing the range of emergent habitat change were again 1958DD and E. Plan 1958DD resulted in a large reduction in area (−75%) and was considered adverse (Table 3) with three other plans with similar outcomes. Plan E was the only plan that provided an increase (26%) in habitat area for emergent vegetation and was considered favorable (Table 3). Two other plans were predicted to result in minor losses in area and were classified as equivocal in impact.

Coastal wetland habitats along the Great Lakes generally develop between the mean and maximum water levels [18]. Wetland plants can survive long periods of flooding, but many require low water levels that expose the substrate for successful germination and seedling establishment [18], [44]. With confined and limited low upslope area at barrier-beach shoreline sites, we assume that increasing the mean water level during the growing season (June–August) would reduce the total wetland area. Prior to regulation of Lake Ontario, the mean water level in a growing season (June–August) was 74.9 m and later the mean for this period increased to 75.0 m. Frenchman's Bay (Ontario), considered here an archetypal barrier-beach site, lost 5.5% of its wetland area [49] under a 0.12 m rise in lake level. Over the water level change of interest, the range of wetland change is expected to be ±5.5% (Figure 4H). Plans 1958DD and B+ show large (6.5%, 5.4%) increases in wetland area and were considered favorable (Table 3). Plan E had a mean loss of 2.5% in area and was classified as adverse. The other plans were predicted to produce minor changes in wetland area and were classified as equivocal.

Great Lakes fish diversity and biomass density are concentrated in nearshore waters, especially in vegetated habitats [50], [51]. The structural complexity of vegetation protects small fish from predation, while abundant invertebrates and high primary productivity provide rich food resources. Thus, a large majority of Great Lakes fishes use protected waters and vegetated habitats for one or more life stages [16]. For baymouth-barrier beach shorelines, we used the northern pike (*Esox lucius*) because it depends on these habitats, has a well documented biology, supports popular fisheries, and is recognized by the public.

Soon after the winter ice clears, northern pike spawn in shallow vegetated waters (typically submerged terrestrial and wetland plants), eggs usually hatch after 12–14 days, and the young remain attached to vegetation for an additional 6–10 days. Young northern pike feed on zooplankton and aquatic insects; thereafter, fish constitute most of their diet [40], [52]. Although spawning habitat is a limiting factor for northern pike reproduction in many waterways, Casselman and Lewis [53] found no relationship between spring water levels and northern pike spawning success. Instead they reported that early life survival played a dominant role in determining the abundance of northern pike in the Bay of Quinte (Ontario). Mortality rates of small young can reach 99% due to stranding and predation [40]. Field studies [54]–[56] reported that pike spawning and early life occurs in water less than 1.0 m deep over newly inundated vegetation with the highest concentration of embryos and new fry in water less than 0.3 m deep. Figure 4I relates a drop in water level to habitat suitability for northern pike embryos and the earliest fry stages during April and May. Embyros are most sensitive to water level changes because of limited mobility and dispersion across depth ranges. Plans 1958DD and 1998 provided excellent (suitability index 0.60) early life habitat for northern pike, and plan E provided the least suitable (0.33) conditions. These plans were classified as favorable and adverse respectively (Table 3). The other plans were intermediate and considered equivocal in impact on northern pike early survival.

The extensive vegetated waters and shores of baymouth-barrier beach sites support a variety of birds. The abundant invertebrates and fish in these habitats constitute a large portion of the diet of many species [57], [58]. Birds are often associated with specific vegetation types [58], [59] with many species preferring an equal mix of emergent vegetation to open water while others favor nesting sites in or near submerged aquatic vegetation [58]. Birds are affected by water level changes both directly, in terms of nest success, and indirectly, through effects on habitat space and food supplies. Taft et al. [60] showed that the average water depth and topographic variability affected foraging habitat availability, which influenced waterbird community composition.

Baymouth-barrier beach shorelines are important to birds that nest at the interface of wetlands and open water. Black tern (*Chlidonias niger*) nest in emergent vegetation and is a New York endangered species [61]. Black terns prefer habitats that have an even mix of open water and vegetation [62] where diverse invertebrates and small fish provide an abundant food supply. Black terns construct their nests on mats of vegetation over water about 0.6 m deep (0.46–1.10 m [41], [63]) from May to August [64]. Eggs are incubated for 17 to 22 days, and young fledge 19 to 25 days after hatching. Rising water levels can result in the loss of nests and young. Figure 4J relates changes in water level during the nesting season with stable water levels being optimal and rises of more than 0.6 m being unsuitable for nesting. Water levels typically increased during the nesting months prior to lake regulation (mean 0.3 m) and the magnitude of increase was greater after lake regulation began (mean 0.4 m). Consequently, all plans resulted in very good nesting suitability (index≥0.66) and considered favorable (Table 3).

Marsh birds construct nests at the interface of open water and plants (emergent, wetland), preferring habitats adjacent to stable shallow water for the nesting season. King rail (*Rallus elegans*) represented marsh birds in this shoreline class; it is endangered in Canada [65], threatened in New York [61], and declining over its range [66]. King rail are found in the Great Lakes region from April to October [67] when they construct nests of dead grasses or sedges in heavily vegetated waters 0.15 to 0.46 m deep [68], [69]. Most eggs are laid from May to June and incubated for 21 to 24 days. Young roam out of the nest to forage (invertebrates and small fish) soon after hatching but they cannot fly for 9 weeks. Water level increases during the nesting season may flood the nests of king rail because they are fixed in low-lying vegetation in water or on vegetated shorelines. Figure 4K shows a relation between habitat suitability and water level increase. Initial increases in water level to common nest heights do not diminish suitability. Further increases result in sharp declines in habitat suitability and additional increases reduce suitable nesting conditions to zero at magnitudes beyond 0.6 m. Water levels have often risen during rail nesting in Lake Ontario prior to regulation and more so afterward, making this species very sensitive to water management plans. All plans except E provided very good nesting suitability (index≥0.71) and considered favorable (Table 3). Plan E was somewhat lower in suitability (0.57) and considered equivocal in effect on king rail nesting success.

### Protected Wetlands

The protected wetlands shoreline class includes tributary mouths, lagoons, and distinct shallow waters with restricted connections to Lake Ontario (Figure 1). Like baymouth-barrier beach shorelines, this shoreline class provides protected, low-energy sites supporting abundant plant growths. Habitats in protected wetlands shoreline class support a large variety of biotic groups, rich plant and animal communities, and many species important ecologically and valued by humans. Protected wetlands are distinct in morphometry and do not move or change shape with changing water levels. A reduction in water level will reduce habitat area and an increase in water level may increase open water and wetland habitats.

Relations between water level change and change in wetlands, emergent plants, and SAV in protected wetland habitats were found to be very similar to those of the baymouth-barrier beach shoreline class. The change in area for SAV growth was estimated using topography of South Sandy Pond; a protected wetland site. Bathymetric measurements were made as described for Blind Sodus Bay, the archetype baymouth-barrier beach site. The resulting relationship between water level and areal change was not meaningfully different from that shown in Figure 4 (F, G, H) because the difference in average underwater slope was very small (0.017 versus 0.024). Therefore, the relations for change in areas of wetlands, emergent plants, and SAV developed for the baymouth-barrier beach shoreline class were used in characterizing water management impacts in the protected wetlands (Table 3).

Some fish species permanently inhabit shallow vegetated habitats that provide refuge from predators, abundant foods, and nesting sites. Bowfin (*Amia calva*) is a resident fish in vegetated habitats. Bowfin spawn from May through June when males prepare a nest in shallow (0.30–0.61 m) vegetated areas. After spawning, the eggs and young are guarded by the male for several weeks (Scott and Crossman 1973). Water level declines during the spawning season may cause eggs and young to be stranded. The minimum water depth for nesting of bowfin is about 0.3 m and we estimate (Figure 4L) a decline in spawning and rearing habitat suitability with rapid water level declines. All water management plans were very similar in providing excellent early life habitat for bowfin: suitability index score from 89 to 98. Therefore, all plans were considered favorable for bowfin in protected wetlands.

Rainbow smelt (*Osmerus mordax*) ascend Lake Ontario tributary streams to spawn in the spring soon after the ice thaws, typically between March and May. Spawning occurs upstream in shallow flowing water locations that tend to be free of vegetation. Eggs adhere to the substrate and hatch after 2 to 3 weeks. Still protected waters associated with tributaries, wetlands, and backwaters provide staging habitats for adults at spawning, and rearing habitat for larvae [40]. The mean water level of Lake Ontario has been 74.81±0.26 m from March through May. With a slope across protected wetlands of 0.017, a 0.26 m rise in mean water level would have flooded approximately 15.24 m at the edge of these habitats. For a small lake tributary with a 1,350 m^2^ open water area at the stream mouth with wetlands, a 0.26 m rise in lake level would be expected to increase open water habitat to 4,056 m^2^ – 300% more assuming a roughly circular water body (Figure 4M). The exact change in open water area will depend on the configuration of a protected wetlands site but this scale of habitat change approximates responses for a range of surface areas. From lake level simulations, most plans were found to substantially (>200%) increase rainbow smelt adult staging and early life rearing habitat and were considered favorable to this fish (Table 3). Plan 1958DD and 1998 providing the smaller gains and were classified as equivocal in changing habitats for smelt.

While various water birds live in protected wetlands of Lake Ontario, we focus on mallards (*Anas platyrhynchos*) to represent effects of water level change. Mallard nests are often constructed in grasses or reeds near water bodies. The mallard dabbles in the shallow water for food, which consists primarily of plants and plant seeds as well as some insects, mollusks, and crustaceans [41], [70]. The loss of wetlands may affect whether mallards are present in an area and will likely influence their nesting success. Both suitable feeding and nesting habitats are needed for supporting mallards. We used a linear combination of SAV and wetland relations (Figure 4F, H) with water level change to capture the cumulative effects on mallards:
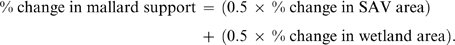



Water level change affects wetlands and SAV in opposite ways so an optimal water level regime for mallards will be a compromise of the two relations. All water management plans provided at least a minor improvement in support for mallards with four plans providing clearly the best conditions (Table 3).

Marsh wren (*Cistothorus palustris*) inhabit marshes almost exclusively. During their breeding season (April through November [71]), marsh wrens are found in marshes and swamps across the northern half of the United States [41]. They use reeds to construct dome-shaped nests about 0.3 to 0.9 m above water [41], [72]. Before water regulation of Lake Ontario, water levels rose an average of 0.66 m during the nesting season and following regulation the seasonal rise was 0.74 m. Changes in habitat suitability for nesting of marsh wren respond to water level increases greater than 0.3 m and declines to zero at 0.9 m (Figure 4N). All plans but E were similar in providing excellent nesting conditions for marsh wren: suitability index score from 89 to 98 and considered favorable (Table 3). Plan E was clearly below the others (79) and classified as equivocal in impact on marsh wren nesting.

Beaver (*Castor canadensis*) is a mammal living in protected wetland habitats and considered is sensitive to lake level fluctuations. Beavers construct lodges at very specific water levels and this species is not normally found in habitats experiencing large fluctuations [73]. Water depth and water level stability are especially important during the winter where ice cover forms. The winter water depth must remain within a range that does not submerge the lodge and remains deep enough for lodge access after ice covers the water surface. Winter water depths between 0.9 and 1.8 m appear necessary for safe beaver lodge sites along the edge of wetlands, tributaries, and land margins. Focusing on water depths for lodges, we use the relation in Figure 4O to estimate effects of water level change on beaver habitat suitability. All water management plans were very similar in providing excellent overwintering conditions for beaver: suitability index score from 93 to 99. Consequently, all plans were rated favorable for beaver habitat Table 3).

### Comparison of Water Management Plans

The ratings of each water management plan on the 20 combinations of representative taxa and shoreline classes (Table 3) show some differences in patterns of impact on the Lake Ontario environment. All water management plans were expected to result in favorable conditions for most representative taxa, and the number of favorable outcomes (8 to 10 out of 16) were similar among all plans. Adverse impacts were most common for plan E and this plan generally set the upper extreme of water levels in regulation simulations (Figure 2). Plan E had five adverse impacts affecting almost all (5 of 6, Table 3) of the vulnerable shoreline classes accounting for about a third (34%, Figure 1) of the lake shoreline. Plan 1958DD had three adverse impacts affecting three of the shoreline classes comprising 15% of the shoreline. This plan closely followed the lower extreme of water levels in plan simulations. The other water management plans were consistently intermediate to plans 1958DD and E in impact ratings and simulated water levels. There were differences in the distribution of favorable and adverse impacts across these five plans but there was little to distinguish these plans in terms of overall effect on the lake environment. The five intermediate impact plans (1958D, 1998, A+, B+, D+) often had equivocal impact ratings (Table 3) and very few adverse impacts. Overall, the natural flow plan (E) is expected to result in the most adverse impact on the current Lake Ontario environment, and current water management (plan 1958DD) has many favorable outcomes and few adverse impacts.

This impact assessment was primarily focused on birds, fish, and plant groups and the water level management plans varied in their impacts by group (Table 3). Plan 1958DD had the most favorable ratings for birds while plan E had clearly more adverse impacts on birds. This pattern reflects the higher water levels, reduced beach area, and faster rising waters during the nesting period under plan E. Fish species that gain habitat under high water levels were favored most often by plan E although changing water levels adversely impacted northern pike. Wetlands were least impacted under plans 1958DD and B+ due to low summer water levels, and emergent and submerged plants were favored under plan E with its relatively high summer water levels. Status quo water management (plan 1958DD) tends to favor birds, fish nesting and early survival, and wetlands. In contrast, plan E tends to favor plants and organisms using shallow aquatic habitats. Other plans were intermediate to these contrasting patterns of impact and difficult to distinguish.

The pattern of impact ratings among shoreline classes (Table 3) was mixed; especially for the most complex classes that offered many habitats (baymouth-barrier beach and protected wetlands). Both favorable and adverse impact ranks were seen within each of these shoreline classes under the distinct plans 1958DD and E. There was some trend for favorable ratings in protected wetlands, and adverse ratings in beach habitats. In general, the pattern of impacts is clearer with taxonomic groupings than shoreline classes.

## Discussion

This ecosystem impact assessment provided results that show similarities and differences among the water management plans relative to select Lake Ontario environmental attributes. The water management plan that emerged from application experience in the last half century, plan 1958DD, appears to be a good choice for maintaining most environmental resources and harming few. This plan had favorable outcomes most often, adverse impacts for half of the shoreline classes comprising a minor portion of the coast, and a long record of successful application experience. Five plans that were designed as modifications to historical management to better address different social and environmental interests slightly reduced the frequency of adverse impacts. However, these plans commonly provide equivocal impact outcomes suggesting many moderately positive and negative effects. It appears that fine-tuning the actual operational plan results in options with many minor impacts of both directions. With little evidence for clear environmental gains from the five refined water management plans a continuation of the current water management policy seems reasonable for the lake environment.

One clear inconsistency with preconceived plan performance was seen with plan E; intended as the natural water level regime with the greatest environment benefits. Instead this plan was most often the one with adverse impacts, impacts on most of the sensitive shoreline classes, and impacts to the largest portion of the lake coast. It appears this natural water management plan would have broad adverse consequences for the lake environment. The belief that plan E is best for the environment rests on the thought that natural hydrologic variation provides the most natural conditions. Plan E was posed as a reference regime for this purpose. The expected changes under plan E may promote past environmental conditions, but in the context of the current lake setting many of these changes would be considered adverse.

The distribution of favorable and adverse impacts across species and assemblages varied without a clear pattern. There was some trend for the current water management (plan 1958DD) to favor species and assemblages that benefit from low, warm season lake levels: inhabitats of beaches, seasonally inundated lands, and stable land-water margins. Plan E, with its high lake levels much of the time, tends to favor inhabitants of shallow water that gain from increased littoral area. Again, the other plans were intermediate to these contrasting patterns of impact and difficult to distinguish. Comparing patterns of impact across water management plans was our assessment aim and the results provide the clearest conclusions when used across plans. Our methods then appear to provide the information that can inform decisions about water management relative environmental resources of most interest.

The management of water in large aquatic ecosystems is expected to result in varied and complex environmental change. Increasingly the management of ecosystem-scale change is being attempted with planning supported by integrated assessments and a systemwide perspective. However, effective integrated assessment practices have not been established, system scale methods are lacking, and the challenges seem overwhelming. For example, management of the Upper Mississippi River-Illinois Waterway was found [74] to be inadequate to support decision-making because it lacked ecosystem scale understanding and broad information synthesis. Similarly, management of water along the Missouri River was reviewed [75] and judged to be very limited by weak consideration of issues at the ecosystem scale, poor responsiveness to public interests, and lack of an ecosystem context for site-specific actions. Ecosystem management requires organizing and synthesizing information at the ecosystem scale while simultaneously addressing public interests, continua and dynamics of physicochemical conditions, and diverse ecological properties. No approach and set of methods will perfectly accomplish integrated ecosystem assessment making the challenge one of selecting a set of tools to present broad, relevant, unbiased, and accessible information to guide decision making. Thus, the product of the assessment needs to be concise but revealing of tradeoffs among policy options.

In structuring our assessment, we made a series of decisions on what to consider, how to encompass the ecosystem, how to consolidate information, and what precision of results are needed for management decisions. Binational government policies, management history, and treaties and laws determined some important attributes of our impact assessment: the management options (water regulation plans), important environmental attributes (species, assemblages), and water level reference data. All large scale ecosystem management cases will have history, law, and precedent that constrain assessment methods and analysis capabilities. We chose a set of impact assessment techniques to fit the situation and meet the need of broad, relevant, unbiased, and accessible information to guide decision-making.

Classification of the Lake Ontario shoreline allowed inferences to be made on how water level change would interact with the biophysical environment. This method provided a whole ecosystem context for analyzing potentially significant water level impacts. A checklist of biotic resources of public interest provided the assignment of what information was relevant in the policy arena. A matrix of environmental attributes and shoreline classes allowed us to infer at a manageable scale which biotic and physical properties needed more detailed analysis. Performance relations integrating water levels and taxon biology enabled us to capture the basic effects of water level management. Analyses were made tractable by working within the concept of representative organisms and archetypal physical settings. Finally, rating impact direction and magnitude diminished the importance of specific numeric predictions (e.g., change in areas) while retaining the capability to compare management options.

Many details of this assessment can be challenged, debated, and improved but as a whole our mix of methods yielded useful findings on the relative merit of seven water management plans for the Lake Ontario ecosystem. The overall outcome was found to be roughly consistent with the ideas used to generate the management plans although they were developed independently of past decisions and management issues. We also draw into question some widely held beliefs: a natural water regime will be best for the environment today, and there are good and bad options for the environment. We do not provide an answer on what is best for the environment although we make a recommendation that one plan appears a reasonable and safe choice. More important, we provide a one page synthesis (Table 3) of information that can inform government officials what environmental tradeoffs are at stake when a plan is considered with other social needs like economic development, transportation efficiency, hydropower, recreation, and shoreline property security.
